# Multimodal skin lesion classification for early cancer diagnosis using deep learning

**DOI:** 10.3389/fphys.2026.1717517

**Published:** 2026-02-09

**Authors:** Vandit Gabani, T. M. Navamani, K. Shyamala, Vinita Kishore Vaswani Rajpal

**Affiliations:** School of Computer Science and Engineering (SCOPE), Vellore Institute of Technology (VIT), Vellore, Tamil Nadu, India

**Keywords:** deep learning, explainable AI, melanoma, pre-trained models, skin cancer, skin lesion classification

## Abstract

**Introduction:**

Skin cancer, particularly melanoma, is a rapidly spreading and potentially life-threatening disease affecting humans. Melanoma typically begins on the skin’s surface before penetrating deeper layers. Early detection significantly improves survival rates, with simple and cost-effective treatments yielding a 96% success rate. Traditional diagnosis methods rely on expert dermatologists, specialized equipment, and invasive biopsies. Deep learning offers advanced solutions for detecting skin cancer earlier and with high accuracy to mitigate costs and assist dermatologists. Deep Convolutional Neural Networks have shown promise in several computer vision tasks, including image classification, prompting their application in dermatology.

**Methods:**

This work focuses on leveraging three prominent DCNN architectures, DenseNet 201, VGG16, and InceptionV3, to classify skin lesions using dermoscopic images. The HAM10000 dataset was taken and divided into training and testing sets. The preprocessing methods include image normalization, scaling, and Otsu’s binary thresholding segmentation and augmentation techniques were applied. We introduced two fine-tuning approaches. Firstly, the top layers of the base model are retrained. Secondly, retraining the half layers of the base models and additional layers are added to form customized CNN models. We merge these underlying models into an ensemble and hyperparameter tuning to enhance performance. The transparency and interpretability of the model are enhanced by Grad-CAM, which raises the model’s dependability for clinical applications.

**Results and Discussion:**

Combining DenseNet-201, InceptionV3, and VGG16, the proposed ensemble model outperforms the individual models with a testing accuracy of 97.9%. Additionally, it exhibits a better F1-score, recall, and precision of 99.2%, demonstrating its efficacy in automated skin lesion detection.

## 1 Introduction

Tumorigenesis, the process by which normal cells undergo genetic alterations, leading to uncontrolled growth, can result in the formation of tumors, both benign and malignant. Malignant tumors can metastasize, while benign tumors typically remain localized. Skin cancer, characterized by the aberrant proliferation of skin cells, represents a pervasive malignancy, with melanoma accounting for a significant portion of diagnosed cases annually. Melanoma’s incidence surpasses that of other common cancers, including those affecting the lungs, bones, and colon. Regrettably, melanoma claims a life every 57 s, underscoring the urgency of early detection for improved prognostic outcomes. Clinicians use advanced imaging techniques like dermoscopy to enhance diagnostic precision and therapeutic efficacy in managing melanoma cases (Gururaj et al., 2023).

Timely prediction plays a vital role in successful treatment; hence, it is essential for individuals to routinely examine their skin and consult healthcare professionals upon detecting any anomalies or alterations. However, even with this understanding, discerning malignant skin lesions remains complex (Saeed et al., 2023). Deep Learning (DL) methods, particularly Convolutional Neural Networks (CNNs), have shown remarkable potential in augmenting diagnostic accuracy. The comprehensive exploration and characterization of DL methodologies for skin cancer diagnosis constitute the focal point of this scholarly inquiry. Their primary goal is to do a thorough review of the literature on deep learning techniques, notably including Generative Adversarial Networks (GANs) tailored for skin cancer diagnostics, alongside Artificial Neural Networks (ANNs), Convolutional Neural Networks (CNNs), and Kohonen self-organizing Neural Networks (KNNs) (Rizvi et al., 2023). Skin cancer, a prevalent malignancy in humans, typically undergoes diagnosis through screening, dermoscopic analysis, histopathological assessment, and biopsy. Physicians often encounter challenges discerning between similar lesions, leading to protracted examinations. Hasan et al. (2023) utilized the EfficientNetsB0-B7 architecture for multi-class skin lesion prediction, leveraging Transfer Learning (TL) and adapting pre-trained weights from the ImageNet dataset. The EfficientNets boast intricate model architectures that necessitate robust technical specifications for efficient processing. Additionally, class imbalance within the ISIC2019 dataset poses a notable challenge, potentially leading to skewed model performance and bias in classification outcomes. Virgens et al. (2025) detected skin cancer at an early stage is particularly difficult due to minor visual variances between non-cancerous and malignant skin lesions; the high amount of similarity among the non-cancerous and malignant types of skin lesions makes it hard for healthcare professionals to classify these lesions. The conventional dermoscope provides limited spectral information that may limit its application as an effective tool for early melanoma diagnosis and has proven not to be as accurate for aggressive types. Although new technologies such as hyperspectral imaging and computational imaging are being developed to enhance the diagnostic capability of healthcare professionals, the expense of these technologies and their associated hardware is limiting their widespread application in clinical practice. Lu et al. (2025) has led researchers to pursue the development of deep learning-based classifiers that can classify skin lesions using traditional dermoscopic images. Deep learning has the potential to give healthcare professionals a valuable resource for improving diagnostic accuracy and enhancing the clinical decision-making process.

Qureshi and Roos (2023) proposed a new CNN architecture that combines pre-trained and data-specific models and metadata to improve classification accuracy. The authors demonstrated that this approach outperforms benchmark approaches on a dataset of 33,126 dermoscopic images. Dandu et al. (2023) concentrated on the segmentation and classification of melanoma skin cancer. Employs a color layout filter model and achieves an impressive accuracy of 90.96%, a precision of 91%, and a recall of 0.91. Also suggests using the efficient attribute-selected classifier, which incorporates image-enhancing techniques, to get better results in melanoma identification. Magdy et al. (2023) suggested two efficient techniques for classifying dermoscopic images using k-Nearest Neighbor and an improved AlexNet model. The authors emphasize the significant contribution of computer vision in early disease diagnosis, particularly in life-threatening situations such as skin cancer. The trials on a dataset of 4,000 images from the ISIC archive highlight the suggested techniques’ superiority over several machine learning and deep learning methods in skin cancer identification. This work proposes a way to automatically classify skin cancer using fine-tuned pre-trained models to address the importance of early diagnosis.

Belmili et al. (2023) used DL methods represented by a two-dimensional Convolutional Neural Network and Multi-layer Perceptron on unimodal and multimodal data. The proposed model classifies images into only two classes: malignant and benign. ResNet152v2, a prominent deep learning algorithm, proves valuable for image classification endeavors. Notably, in the context of the HAM10000 dataset, model development encountered challenges stemming from class imbalance, leading to overfitting issues. Despite these hurdles, the achieved accuracy is 86% (Korsak et al., 2023). To mitigate these issues, Computer-Aided Diagnosis (CAD) systems employing deep learning techniques offer a promising avenue by automating lesion detection and classification processes. Melanoma, identified as the deadliest form of skin cancer, is witnessing a concerning surge worldwide, underscoring the urgency for precise and efficient diagnostic methodologies (Ichim and Popescu, 2020). Bijoy et al. (2025) presented SkinIncept, an ensemble CNN model combining six pre-trained networks (including InceptionV3 and Inception-ResNet-V2) for classifying ten common skin diseases. The authors performed extensive preprocessing and reported the ensemble reaching 96.52% accuracy. The authors demonstrate the power of transfer learning and ensembling multiple CNN architectures to improve classification performance, and show how tuning (ablation studies) can fine-tune such models. Chatterjee et al. (2024) proposed IncepX-Ensemble, combining InceptionV3 and Xception via transfer learning on the HAM10000 dermoscopy dataset. They tackled class imbalance by data augmentation. On a balanced HAM10000 set, the IncepX-Ensemble achieves about 98% training and test accuracy.

Ieracitano et al. (2025) introduced a CNN-based classifier (EfficientNet-B0) for classifying melanoma, nevus, and keratosis from the ISIC dermoscopy dataset and critically defined a novel Trustworthiness Index (TIxAI) for explainable AI. TIxAI measures the difference in relevance between lesion and non-lesion regions in Grad-CAM maps, arguing that a higher index means more trustworthy predictions. The authors provided a concrete method to quantify explainability in skin lesion models. Also, the proposed model’s relevance lies in its focus on XAI: it shows how to evaluate and improve the interpretability of deep models, directly supporting the literature on explainable diagnostics in dermoscopy. Dakhli and Barhoumi (2024) quantitatively compared four *post hoc* explainability methods (LIME, SHAP, attention maps, and Grad-CAM) for skin-lesion CNNs (Inception-ResNetV2). The authors generate occluded images based on saliency maps and measure prediction change as a faithfulness metric. The SHAP-based explanations are the most faithful, outperforming Grad-CAM, LIME, and attention maps. The authors proposed a robust deep learning framework for skin lesion classification, addressing key research gaps. The model combines DenseNet201, VGG16, and InceptionV3, fine-tuned using a hybrid strategy. Incorporates augmentation techniques to mitigate class imbalance. Grad-CAM provides transparent visual explanations for each classification, enhancing interpretability and clinical applicability. Hence, we propose a skin lesion classification for early cancer prediction using deep learning models to address these research gaps.

The main contributions of this work are as follows:

- We propose an automated skin lesion classification system that utilizes pre-trained DCNNs and interpretability for decision-making. This system could significantly aid dermatologists in early detection and treatment.
- The incorporation of pre-trained and ensemble model enables high accuracy by adding layers to the pre-trained models in skin lesion classification.
- Exploring the impact of CNN architecture variants and hyperparameter tuning on transfer learning performance for skin lesions.
- Conducted a comparative analysis of three prominent DCNN architectures (DenseNet201, VGG16, and InceptionV3) for effective skin lesion classification with existing works.

This study presents a multi-dimensional framework for skin lesion classification that incorporates (i) a dual-phase fine-tuning strategy to enhance feature extraction and model generalization, (ii) a comparative analysis of hybrid training methodologies involving selective retraining and batch normalization adaptation, (iii) an ensemble model architecture that integrates DenseNet201, VGG16, and InceptionV3 to bolster classification robustness, (iv) class-specific weighted loss functions to mitigate imbalance in dermatological datasets, and (v) the utilization of eXplainable AI techniques (Grad-CAM) to augment model transparency and clinical trust. This article jointly tackles both technical and clinical problems in AI-enhanced dermatological diagnosis.

The following sections of this work are structured as follows: Section 2 presents a literature review, examining prior research on skin lesion classification models utilizing various DL techniques. Section 3 delves into the proposed approach for the skin lesion classification task. Section 4 presents the results and model evaluation metrics, followed by subsection A for a comparative analysis of different model performances. We discuss the advantages and limitations of the proposed model presented in Section 5. Finally, Section 6 summarizes the research’s key findings and provides concluding remarks regarding the proposed skin lesion classification model.

## 2 Related works

Deep Learning (DL) in healthcare is becoming increasingly popular in this rapidly evolving era. Early diagnosis of multimodal skin lesions has been the subject of extensive research. In the study by Gururaj et al. (2023), skin cancer was portrayed as a rapidly increasing global health concern, emphasizing early detection to implement adequate preventive measures. DL methodologies, notably Convolutional Neural Networks (CNNs), have emerged as promising tools for object detection and classification tasks, thus offering considerable potential in skin cancer diagnosis. The research leveraged the MNIST: HAM10000 dataset, encompassing seven distinct categories of skin lesions, and employed data preprocessing techniques like sampling and segmentation facilitated by autoencoders. Their study utilized transfer learning strategies for model training, specifically DenseNet169 and ResNet50 architectures. Ichim and Popescu (2020) presented an intelligent system that used two levels of prediction for melanoma detection. In the first level, five classifiers, including perceptrons coupled with color features, a generative adversarial network for segmentation, ResNet, and AlexNet for analyzing various characteristics of lesions. These classifiers fed into a second-level perceptron that made the last decision on melanoma presence. The system achieved a precision of 97.5% and an F1 Score of 97.47%, surpassing previous literature. Kassem et al. (2020) suggested a model for accurately classifying skin lesions using transfer learning techniques. The model attained a classification accuracy of 94.92% on the ISIC 2019 dataset, which comprises eight lesion classes. Furthermore, the study proposed a method to mitigate class imbalance, enhancing performance metrics.

Nigar et al. (2022) introduced a skin lesion classification system based on Explainable Artificial Intelligence (XAI) to improve accuracy and interpretability in early skin cancer diagnosis. Using the ISIC 2019 dataset, the model achieved an accuracy of 94.47% in classifying eight types of lesions. The incorporation of explainability enhances the model’s utility in clinical settings. Sorour et al. (2023) presented a dermatology detection system that utilizes Deep Learning (DL) and object recognition techniques. The system consists of three phases: data preprocessing, data augmentation, and prediction with localization. Experimental findings showcase enhanced accuracy, especially in predicting vitiligo and melanoma, surpassing recent methodologies. Wei et al. (2020) introduced a lightweight skin cancer recognition model to improve prediction accuracy while minimizing model complexity. The model incorporates two feature extraction modules: a Lightweight CNN for lesion classification and a feature discrimination network. This approach improves lesion recognition and segmentation capabilities while reducing model parameters, demonstrating its practical potential in dermatology applications.

Ahmed et al. (2024) presented a deep learning-based approach for melanoma skin cancer detection, utilizing a stacked Convolutional Neural Network (CNN) architecture. Comparative analysis with pre-trained CNN variants (VGG16, Inception V3, ResNet50, EfficientNetB0) and traditional machine learning algorithms (Logistic Regression, Support Vector Classifier, Random Forest, Gradient Boosting Machine, K-Nearest Neighbor) demonstrated the superior performance of the model, attaining a testing precision of 96% and a validation accuracy of 73%. Notably, overfitting was observed due to a significant disparity between the accuracy of the training and validation datasets.

Surendren and Sumitha (2023) conducted a comparative analysis of five traditional machine learning algorithms: Decision Tree, K-Nearest Neighbors (KNN), Naïve Bayes (NB), Logistic Regression (LR), and Support Vector Machine (SVM) for skin cancer diagnosis. Utilizing the ISIC database comprising 2,600 training images and 650 test images, SVM emerged as the top-performing classifier, demonstrating superior accuracy. The study emphasizes the importance of AI algorithms complementing the expertise of healthcare professionals rather than replacing it, facilitating decision-making and enhancing patient care.

Ahmed et al. (2023) investigated the need for Computer-Aided Diagnosis (CAD) systems capable of automatically extracting distinctive features for accurate skin cancer prediction, a prevalent and severe malignancy. Utilizing images from the ISIC dataset, the researchers employed CNN, SVM, KNN, and Naive Bayes algorithms, with CNN exhibiting the highest accuracy of 100% on the test set. Swathi et al. (2023) performed a comparison investigation to assess the efficacy of pre-trained models in early skin cancer diagnosis. Engaging renowned CNN variants such as VGG16, InceptionV3, and EfficientNetB0 on the Integrative Skin Imaging Collaboration dataset, the study achieved an accuracy range of 80%–83%. However, the research identified a need for improved interpretability in these models, suggesting opportunities for future enhancements.

The existing research works face several challenges: there’s a notable variation between training and validation accuracies, limiting the model’s generalization; they often focus solely on predicting benign or malignant classes rather than all other classes in the HAM10000 and ISIC2019 datasets; VGG16 and ResNet exhibit low accuracy, around 74.8%, and 80% respectively. While EfficientNet is utilized, it still struggles with an accuracy of around 95%, with inconsistent validation results, and lacks segmentation; some studies rely on small datasets containing only 2,000 to 3,000 images, impacting model performance; although deep learning algorithms achieve 87% accuracy, there’s room for improvement. Deep learning has demonstrated significant potential in transforming the process of diagnosing skin cancer. These models can assist dermatologists in promptly identifying and treating them by efficiently classifying skin lesions. Although pre-trained models have demonstrated significant achievements, there is a distinct requirement for customized architectures designed explicitly for skin image analysis. Furthermore, it is imperative to improve the comprehensibility of models and tackle issues like data imbalance, feature extraction, and interpretability (Shah et al., 2023) to facilitate the extensive utilization of DL models in clinical environments.

Thwin and Park (2024) combine three cutting-edge CNNs (VGG16, ResNet-50, and Inception-V3) to create a skin lesion categorization system. The outputs of each model are combined using weighted averaging after they have been pretrained on ImageNet and refined on dermoscopic images. With an accuracy of approximately 91% on the original ISIC dataset and approximately 97% on a balanced, this ensemble routinely beats the individual networks. Their ensemble also achieves 90%–96% accuracy on the HAM10000 dataset, proving that integrating multiple CNNs improves classification performance by capturing a variety of lesion properties. Similarly, Selvaraj et al. (2024) used a number of sophisticated network designs in an ensemble approach. Using skin lesion images, they train models like ResNeXt101, SE-ResNeXt101, ResNet152V2, DenseNet201, GoogLeNet, and Xception using stochastic gradient descent. Class imbalance and noise are addressed by creating both “average” and “weighted” ensembles of these models’ outputs. Individual models are greatly outperformed by the ensemble models: the weighted ensemble achieves 97%, while the average ensemble achieves 96% macro-ROC-AUC. According to this study, combining several different deep networks might increase diagnostic accuracy. Some pieces combine several feature representations into one framework. A dual-track deep network that records both local and global context is proposed by Ramamurthy et al. (2024). One branch is a customized CNN with a Feature Pyramid Network (FPN) and Global Context Network (which captures multi-scale and global information), and the other is a modified DenseNet-169 with a coordinate attention module (which extracts fine-grained, local features). The feature maps of these branches are then merged for categorization. With an accuracy of 93.2% on HAM10000, this integrated design surpasses earlier single-branch models.

In the same way, Hasan et al. (2022) present DermoExpert, a hybrid CNN architecture that processes each image using three feature-extraction modules in tandem. These modules’ feature maps are combined and supplied into distinct classification heads, each of which makes an ensemble prediction. Additionally, DermoExpert uses class-rebalancing, data enrichment, and lesion segmentation in its preprocessing. The advantages of merging several feature extractors are demonstrated by the extremely high performance (AUC 0.96–0.97 on ISIC-2016/17/18 datasets) produced by this multi-branch ensemble. Su et al. (2024) use a unique GAN-based method to address data imbalance. Their Self-Transfer GAN (STGAN) creates realistic images by first learning aspects of general lesion images and then fine-tuning to each class. The training set is enhanced by the artificial visuals. The methodology produces state-of-the-art performance on HAM10000 (e.g., 98.23% accuracy, 89.5% F1-score) when combined with a ResNet50-based classifier (with test-time augmentation). Because it enhances both majority and minority classes, the STGAN has a strong ensemble-like effect and increases sample diversity (higher FID and Inception scores). This demonstrates how combining classification and generative modelling may greatly increase the accuracy of skin lesion diagnosis. A realistic, single-model pipeline tailored for edge devices is highlighted by Hoang et al. (2022). To separate lesions, they suggest an entropy-weighted FCM segmentation. A bespoke wide-ShuffleNet CNN is then used for classification. Although their solution is lightweight enough for mobile use, it delivers more accuracy than previous attempts on HAM10000 and ISIC2019. The new segmentation phase in this design enhances the quality of lesion detection, which increases the classification accuracy of the single ShuffleNet model. Although this method deviates from the ensemble trends mentioned above, it shows how segmentation and CNN can be combined in a different way to provide robust lesion classification. Instead of depending on a single CNN, the goal of all these investigations is to improve skin lesion categorization by mixing several models or approaches. They show that skin cancer may be diagnosed from images with greater accuracy and resilience using ensemble techniques, multi-branch architectures, or integrated data augmentation/classification pipelines. Hence, to address these issues, we propose a system for enhancing the prediction of multi-modal skin lesions for early cancer diagnosis.

## 3 Materials and methods

**FIGURE 1:**
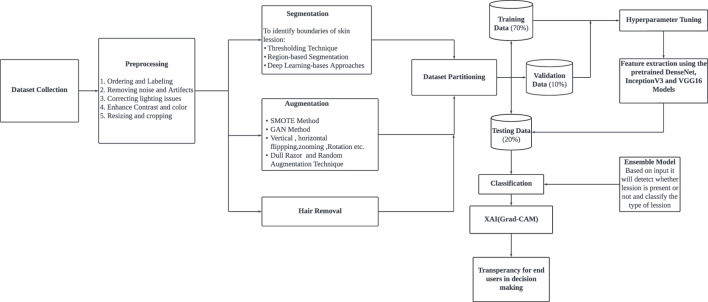
Architectural diagram of the proposed system.

The proposed methodology encompasses several stages: data collection, preprocessing, segmentation, augmentation, feature extraction, prediction, and classification. Figure 1 shows a detailed overview of the proposed model. This approach comprises a series of steps for processing and analyzing a dataset to build an accurate and reliable deep-learning model. The process begins with preprocessing, which involves organizing and labeling the dataset, eliminating noise artifacts, enhancing contrast, addressing lighting issues, resizing images, and other necessary adjustments. Following this, Feature Extraction is conducted to streamline the model by identifying crucial features while discarding less significant ones. Model training entails building a series of classification models, addressing class imbalance through segmentation, augmentation, and oversampling, and employing cross-validation for performance assessment. Finally, Model evaluation is carried out by comparing various models using metrics like precision, recall, F1 score, confusion matrix, and classification report.

### 3.1 Dataset description

Numerous publicly accessible datasets are often used for skin lesion research, including ISIC2017, ISIC2018, ISIC2019, ISIC2020, BCN20000 and HAM10000. This work uses the HAM10000 dataset, which provides a broad collection of dermoscopic images depicting pigmented skin lesions. These include actinic keratoses, basal cell carcinoma, benign keratosis-like lesions, dermatofibroma, melanoma, melanocytic nevi, and vascular lesions. The dataset comprises approximately 10,000 high-quality photos, mostly histopathologically validated, making it a solid and dependable resource for training and assessing deep learning models in dermatological diagnosis.

Other cases are validated through follow-up examinations, expert consensus, or *in-vivo* confocal microscopy. Each lesion may feature multiple images, facilitating tracking through the lesion_id column in the metadata file. Table 1 shows a detailed dataset description with a count of the total images for each class. The HAM10000 dataset comprised seven distinct skin lesion classes and was utilized for training, testing, and validating the model. It comprised a total of seven distinct skin lesion classes. **nv**: Melanocytic nevi.

**TABLE 1:** Dataset counts.

Dataset	Classes	Training	Testing	Total
HAM10000 (7 classes)	Melanocytic nevi	4,690	2,015	6,705
	Melanoma	770	343	1,113
	Benign keratosis	700	399	1,099
	Basal cell carcinoma	357	157	514
	Actinic keratosis	227	100	327
	Vascular lesions	100	42	142
	Dermatofibroma	81	34	115
	**Total**			**10,015**

Bold values indicate the best-performing results or key highlighted values in each table for easy comparison.

**Mel**: Melanoma

**bk**: Benign keratosis-like lesions

**bcc**: Basal cell carcinoma.

**Akira**: Actinic keratoses

**vasc**: Vascular lesions

**df**: Dermatofibroma. Since HAM10000 contains multiple images per lesion, we used the metadata lesion_id to enforce a lesion-wise split. All images belonging to the same lesion were assigned to a single partition (training, validation, or testing) to prevent data leakage. The dataset does not provide a consistently reliable patient identifier; therefore, lesion-wise grouping represents the strictest leakage-free protocol supported by the dataset. In addition, we report Group K-Fold cross-validation results (mean ± 95% confidence interval) using lesion_id as the grouping variable to ensure robust and unbiased performance estimation.

### 3.2 Preprocessing

**FIGURE 2:**
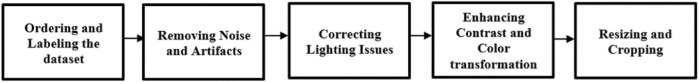
Preprocessing steps.

The preprocessing section describes the procedures used to optimize feature extraction, ensure consistency, and improve image quality for precise skin lesion classification. Incorporates segmentation, augmentation approaches, noise reduction, lighting correction, dataset structure, and image scaling to maximize model performance. The preprocessing consists of different subparts, as shown in Figure 2. To ensure that all dermoscopic image samples were the same size and can be used together without excessive processing time, each image was downsized to 100 × 75 pixels. These dimensions were used because they provide enough detail regarding a lesion’s shape, colour distribution and coarse texture while allowing for efficient training of ensemble-based deep learning architectures in resource-limited settings.

Several crucial phases are included in the preparation procedures to improve image quality and ensure dataset consistency. The dataset consists of columns like lesion ID, image ID, lesion class (dx), diagnostic type, age, sex, and location, which are included in organizing and labeling the dataset. Columns were added to the dataset such as:

- path to store the local path for each image.
- cell_type to store the full name of dx (class of lesion).
- cell_type_idx to store a unique code for each dx.
- label to map values in the dx column to integer labels using the dictionary.

**FIGURE 3:**
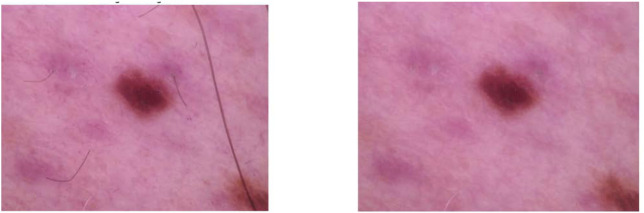
Image before and after applying hair removal.

To ensure consistency across the dataset, images are downsized using the NumPy library, which transforms them into NumPy arrays and standardizes their dimensions to 100 × 75 pixels. Gaussian filtering smoothes visual distortions while maintaining important lesion details by eliminating image noise and artifacts. Histogram equalization methods address lighting discrepancies, improving image quality by adjusting brightness fluctuations and enhancing contrast. Additionally, color alterations improve image representation, while adaptive contrast enhancement algorithms selectively modify image contrast depending on local variables, maintaining essential elements. Hair removal is also done using Gaussian filtering to remove undesired artifacts that might impede lesion categorization. The input image is loaded, converted to grayscale, and then subjected to morphological processes to identify and eliminate hair strands. The widely used (Güler and Ağraz, 2025) method is utilized as a pre-processing step to remove hair from images, as shown in Figure 3. By optimizing the dataset for reliable feature extraction, these preprocessing processes improve the accuracy and generalization of the model. In this study, we improved the image quality for skin lesion classification by implementing a strong preparation pipeline modeled after the DullRazor method and making use of contemporary OpenCV capabilities. In order to eliminate high-frequency noise, especially salt-and-pepper artifacts, while maintaining crucial structural elements like lesion edges, Gaussian filtering was first used. With a kernel size of (3 × 3) and σ = 0.5, we were able to effectively balance denoising and edge retention using OpenCV’s cv2.GaussianBlur(), which is essential for downstream classification accuracy. Histogram equalization was used to improve contrast after noise reduction. This was accomplished by converting the image to the YUV color format and using cv2.equalizeHist() to equalize the Y (luminance) channel, making sure that the lesion’s color and texture were preserved and that only brightness levels were changed. After that, the improved image was transformed back into BGR format. By ensuring better visibility of lesion boundaries and subtle features, this two-step preprocessing noise reduction, followed by contrast enhancement, helps the classifier differentiate between various skin lesion types more accurately.

### 3.3 Segmentation and augmentation

Segmentation is the process of isolating regions of interest for study, whereas augmentation improves a model’s performance by artificially increasing the size of the dataset.

#### 3.3.1 Segmentation

**FIGURE 4:**
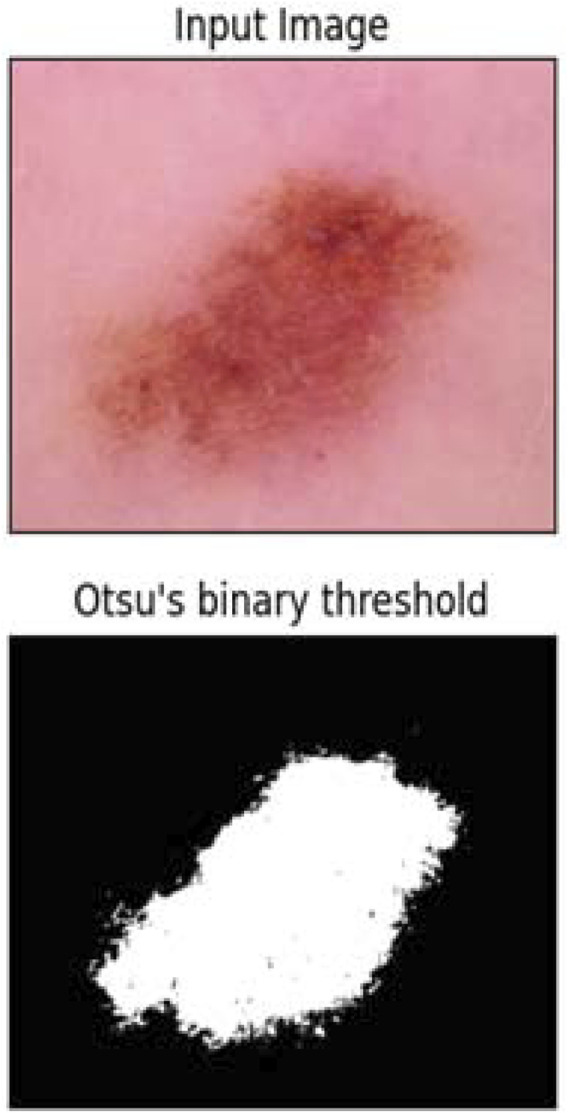
Otsu’s binary thresholding segmentation. The input image (top) is segmented into a binary mask (bottom), highlighting the lesion by automatically selecting an optimal threshold.

Segmentation played a crucial role in identifying and delineating the boundaries of skin lesions within images, enabling accurate analysis and diagnosis. Various segmentation algorithms, such as thresholding techniques, region-based segmentation, and deep learning-based approaches, were explored. In this work, both thresholding-based and region-based segmentation techniques were investigated. The thresholding-based method yielded superior results compared to the region-based approach. Specifically, Mridha et al. (2023) binary thresholding approach was employed for segmentation. Otsu’s binary thresholding is a segmentation technique to distinguish regions of interest from the background in an image. It automatically computes the optimal threshold value by examining the histogram of pixel intensities, aiming to enhance the differentiation between foreground and background classes, as shown in Figure 4. Otsu’s method effectively generates a binary mask by selecting a threshold that minimizes intra-class variance and maximizes inter-class variance. This mask designates pixels with intensity values surpassing the threshold as foreground, while those falling below are categorized as background. The supplied image is first transformed to grayscale, making the lesion look darker and the backdrop brighter. The foreground (lesion) and background are represented by two peaks in the produced pixel intensity histogram. By optimizing inter-class variance, Otsu’s technique establishes an ideal threshold that successfully isolates the lesion from the background. By strengthening lesion borders, this segmentation stage improves feature extraction for classification. Although Otsu’s thresholding method provides a basic yet effective means for quickly and accurately determining where the lesions are located in an image with no or little computational load. Since segmentation is only used as part of pre-processing, the authors wanted to use a more efficient method rather than using complicated deep learning models for the segmentation process.

#### 3.3.2 Augmentation

Data augmentation played a crucial role in multimodal skin lesion classification by increasing the size and variety of the training dataset, thereby enhancing the agility and generalization of classification models. Standard augmentation methods included image rotation, translation, scaling, flipping, and noise addition, introducing variations to the data and improving the model’s adaptability to diverse scenarios. Additionally, addressing class imbalance was essential, with techniques like SMOTE and GANs used to generate synthetic samples for minority classes, thereby balancing the dataset. Tailoring augmentation strategies to skin lesion characteristics further refined the model’s ability to identify various lesion types. Overall, data augmentation enriched the training data effectively, mitigating overfitting and enhancing the model’s performance on real-world datasets. As illustrated in Table 1, the dataset exhibits imbalance, particularly biased towards Melanocytic nevi lesion types, comprising nearly half of the dataset. A maximum limit of 3,500 images per class was established to address this, resulting in a total dataset size of 24,500. All artificial samples generated through augmentation were used exclusively for training, while the validation and test sets comprised only real, non-augmented HAM10000 images.

For classes exceeding 3,500 images, a random selection process retained 3,500 images while discarding the rest; however, classes with fewer than 3,500 images required oversampling through augmentation techniques. Various methods, including GAN, SMOTE, and random augmentation techniques like rotation, vertical flip, horizontal flip, shear, zoom, height shift, and width shift, were employed to balance the dataset and enhance across all classes, as shown in Table 2. When augmentation and segmentation approaches are combined, lesion localization improves, dataset variety increases, and the model’s accuracy and resilience increase. Image-level and feature-level augmentation strategies were utilised to mitigate class imbalance. A DCGAN architecture was employed for image synthesis, featuring a transposed-convolution generator (ReLU, batch normalisation) and a convolutional discriminator (LeakyReLU). The DCGAN underwent training for 100 epochs utilizing the Adam optimiser (learning rate = 0.0002, = 0.5). Furthermore, SMOTE was utilised on the extracted feature representations to augment minority classes. All synthetic data were utilised solely for training, whereas the validation and testing sets comprised exclusively real HAM10000 images.

**TABLE 2:** Class-wise distribution of the HAM10000 dataset before and after data augmentation.

Skin lesion class	Training (real, before Aug.)	Testing (real)	Total real samples	Training (after augmentation)
Melanocytic nevi (nv)	4,690	2,015	6,705	3,500
Melanoma (mel)	770	343	1,113	3,500
Benign keratosis-like lesions (bkl)	700	399	1,099	3,500
Basal cell carcinoma (bcc)	357	157	514	3,500
Actinic keratoses (akiec)	227	100	327	3,500
Vascular lesions (vasc)	100	42	142	3,500
Dermatofibroma (df)	81	34	115	3,500
**Total**	**6,925**	**3,090**	**10,015**	**24,500**

Bold values indicate the best-performing results or key highlighted values in each table for easy comparison.

Algorithm 1 **Workflow for Classifying Skin Lesions**

1: **Step 1: Data Acquisition**
2: Import and structure the HAM10000 dataset.
3: Include additional columns for image paths, lesion class names, unique codes, and integer labels.
4: **Step 2: Preprocessing of Data**
5: Remove noise and imperfections.
6: Address illumination inconsistencies.
7: Enhance brightness contrast and apply color transformations.
8: Utilize DullRazor to resize images and remove unwanted hair.
9: **Step 3: Data Augmentation**
10: Balance class distribution with a maximum of 3,500 images per class.
11: Apply augmentation techniques such as rotation and flipping.
12: Utilize SMOTE and GANs for minority class oversampling.
13: **Step 4: Model Development**
14: Perform feature extraction using pre-trained models.
15: Optimize pre-trained models (DenseNet201, VGG16, InceptionV3).
16: Design and implement a Custom CNN architecture.
17: **Step 5: Model Training**
18: Train each base model using fine-tuning techniques.
19: Develop an ensemble model by combining all base models.
20: Optimize hyperparameters for improved performance.
21: **Step 6: Model Evaluation**
22: Evaluate models using accuracy, recall, F1-score, and support metrics.
23: Perform cross-validation for model comparison.
24: Analyze results to select the best-performing model.

### 3.4 Proposed model design

This phase entails the sequence of processes to enhance skin lesion prediction. Algorithm 1 shows the workflow for classifying skin lesions.

**FIGURE 5:**
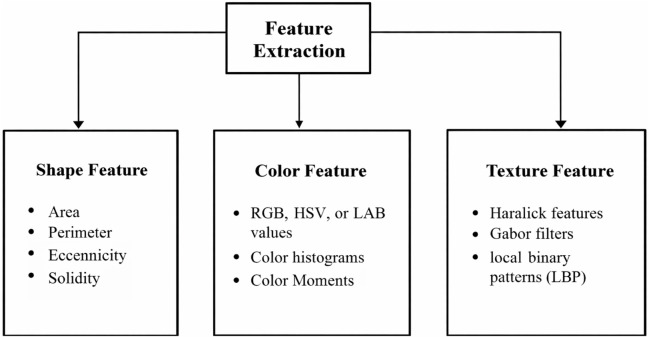
Feature extraction.

**FIGURE 6:**
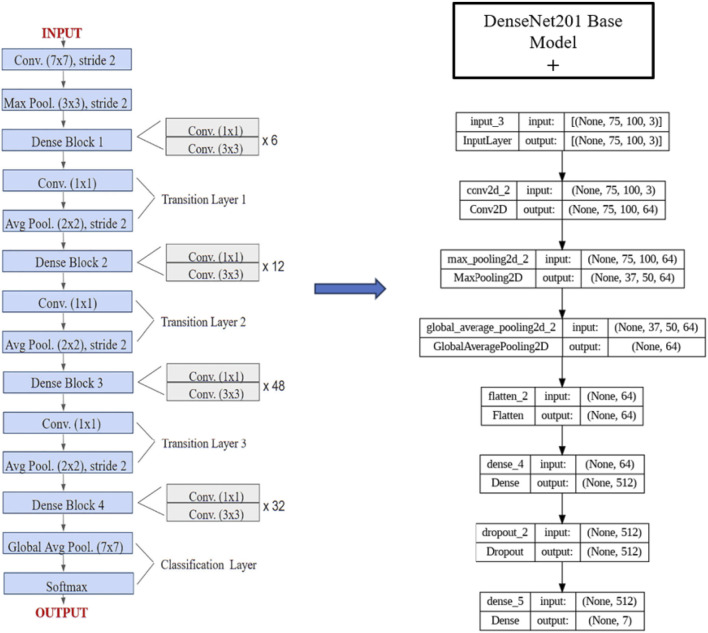
DenseNet-201 base model architecture Jaiswal et al. (2021) and Proposed DenseNet201 Model.

The ensemble model integrates the outputs of DenseNet201, InceptionV3, and VGG16 using a technique known as soft voting through averaging. By calculating the element-wise average of their output probability distributions, the predictions from each separate model are integrated in this method. The layers are used to achieve this strategy. In actual use, the ensemble model first independently runs the input data through DenseNet201, InceptionV3, and VGG16, the three foundation models. The predictions produced by each of these models are usually expressed as sigmoid or softmax probability vectors. The final predicted probability for each class label is the arithmetic mean of the corresponding probabilities from all three models once these individual outputs are gathered and averaged collectively. This averaging ensures that the ensemble decision reflects a consensus among the models, reducing the impact of any single model’s error. This soft voting method is straightforward but successful. It makes the assumption that all models are equally reliable and does not call for training a second classifier on top of the basic models. Utilizing the advantages of several architectures provides a fair and computationally effective way to increase overall prediction resilience, even though it might not be able to capture intricate inter-model interactions as stacking or weighted voting would. In the model training phase, 70% of the dataset was used as the training set. To enhance data quality and optimize model performance, a feature selection process was conducted to identify the most significant features based on their importance. Subsequently, these selected features were retained for model training, while less impactful features were excluded from the dataset. This meticulous feature selection approach aimed to streamline the dataset, focusing the model’s learning process on the most relevant information to improve its predictive capabilities and overall performance, as shown in Figure 5. Different base models, such as DenseNet 201, VGG16, and InceptionV3, were used to train the model. Three approaches were employed for fine-tuning the model: firstly, the base model was trained by retraining only the top layers, which did not yield satisfactory accuracy; secondly, a custom CNN model was created and trained; and thirdly, a model was developed by retraining half of the layers of the base models and adding CNN layers. Figure 6 depicts the architectural design of the DenseNet201 base model and the proposed DenseNet201 model. The base model is a convolutional network known for dense connections. The base model delineates the data flow across the network, emphasizing relevant features and their linkages. The proposed model consists of the base model, and additional layers are added to predict skin lesions. In the proposed work, our initial approach involves retraining the upper layers of the base models to classify skin lesions. However, we discovered that this method did not lead to an improvement in accuracy.

**FIGURE 7:**
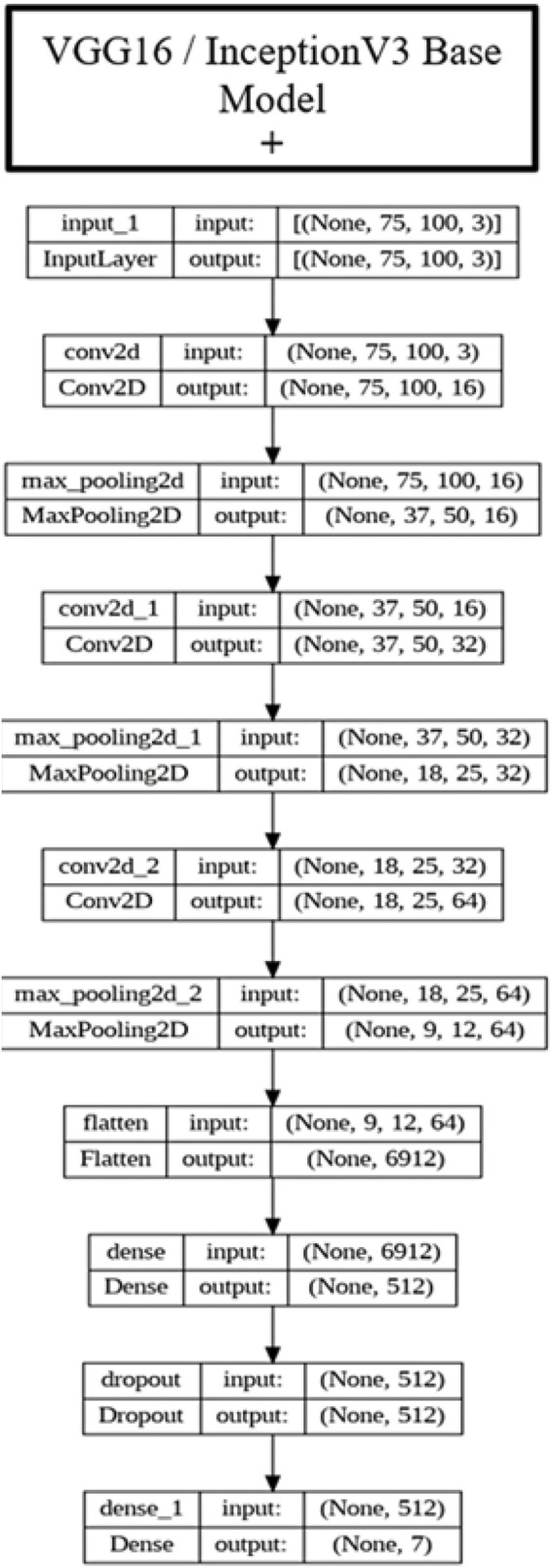
Proposed VGG16 and InceptionV3 (fine-tuned VGG16 and InceptionV3).

The base model’s layers were adjusted to improve accuracy. Fifty percent of the layers were frozen, while the remaining were retrained. In addition, further convolutional layers were included in these foundational models, as seen in Figures 6, 7. Due to the intricate nature of DenseNet201, a reduced number of Convolutional Neural Network (CNN) layers were incorporated compared to the other two foundational models. The DenseNet201 architecture incorporates supplementary layers, starting with an input layer designed for images of 75 × 100 pixels and including 3 color channels. This is followed by a convolutional layer that recovers 64 feature maps. The features underwent downsampling via max pooling, which decreased the spatial dimensions while maintaining the depth. Subsequently, a global average pooling layer was employed to compress each feature map into a singular value, followed by a flattening layer. A deep layer containing 512 units was used to effectively capture intricate patterns, while dropout was implemented to mitigate the risk of overfitting. The last dense layer generated 7 output units corresponding to the classification task’s distinct classes. In both InceptionV3 and VGG16 models, the CNN architecture included the following layers: input images of dimensions 75 × 100 with 3 color channels were passed through three convolutional layers, with the number of filters gradually rising from 16 to 64. After each convolutional layer, the max-pooling layers further decreased the spatial dimensionality. Following the last convolution and pooling procedures, the feature maps were transformed into a one-dimensional vector with a size of 6,912. The vector was subsequently fed into a thick layer with 512 units to facilitate the acquisition of intricate patterns. Additionally, a dropout layer was used to mitigate the risk of overfitting. The output of the last thick layer consisted of 7 units, each corresponding to a specific categorization category.

Incorporating CNN layers into the existing base models yielded advantages. It facilitated the retrieval of more intricate and sophisticated characteristics from the images, improving the model’s ability to discern minor distinctions among different skin lesions. Consequently, this enhancement improved the precision of the models, making them more resilient and efficient in categorizing various skin lesions. Figure 7 depicts a proposed VGG16 and InceptionV3 with fine-tuning. The proposed model contains a base model of the VGG16 and Inception V3, and additional layers are added. The network is comprised of several convolutional and max-pooling layers that are used to extract information from the input image. Next, the feature maps that have been retrieved are flattened and input into thick layers using dropout, which helps with regularization. The ultimate output layer generates seven numbers, likely representing the probabilities of various classes in a classification. Contrary to the uniform stack of smaller filters in VGG16 or the complicated parallel processing modules in Inception V3, the proposed design seems more straightforward. To conduct a more conclusive comparison, it is necessary to obtain comprehensive data on the sizes of the filters, the activation functions used, and the total depth of the network. Figures 6, 7 illustrate the base model architectures for DenseNet 201, VGG16, and InceptionV3. The base model layers were modified by freezing half of the layers and retraining the remaining half of the layers. Additionally, extra layers were incorporated into these base models. In fine-tuning, higher-level layers were retrained while early layers were frozen: VGG16 (Blocks 4–5), InceptionV3 (Mixed7–9), and DenseNet-201 (Dense Blocks 3–4).

### 3.5 Model training

Initially, the base models were loaded using a library. Subsequently, the top layers of each base model were retrained by setting “layer. trainable = True.” This process was repeated for each base model, including DenseNet201, InceptionV3, and VGG16. Following this, a custom model was created comprising the following components:

$$\begin{array}{cc} & \text{InputLayer}\to \text{Conv}\times 2\to \text{MaxPool}\to \\ & \text{Dropout}\to \text{Conv}\times 2\to \text{MaxPool}\to \\ & \text{Dropout}\to \text{Flatten}\to \text{Dense}\to \\ & \text{Dropout}\to \text{Dense} \end{array}$$

The models were trained on the same dataset. Following this, an attempt was made to train the base model by retraining half of its layers while adding additional layers. Considering DenseNet201’s complexity, fewer layers were added compared to the other models. Subsequently, an ensemble model was created by combining these three models. Additionally, various hyperparameter tuning methods were implemented to optimize the models, as depicted in Table 3. This study aims to balance accuracy and computational cost to improve DL models for skin lesion categorization. To preserve feature details and increase processing efficiency, input dimensions of 75 × 100 × 3 were used, and hyperparameter adjustment was essential. Training was carried out for 50 and 120 epochs to examine the trade-off between underfitting and overfitting, and a batch size of 64 was used to optimize GPU memory usage.

**TABLE 3:** Hyperparameter tuning.

Parameter	Value
Input size	( 75×100 × 3)
Batch size/epochs	64/(50, 120)
Loss function	Categorical cross-entropy
Output activation function	Softmax
Activation function	ReLU
Optimizer	Adam
Learning rate	0.00001
Momentum	0.9
Dropout	0.5
Training/testing/validation split	70%/20%/10%

The loss function was categorical cross-entropy, and the softmax activation function guaranteed the probability distribution across many classes. Hidden layers used the Rectified Linear Unit (ReLU) to speed up convergence and avoid vanishing gradients. The Adam optimizer was selected for its momentum-based flexibility and effective learning rate modifications, and a modest learning rate of 0.00001 was used. After training each model using customized pre-trained models, the models were saved in Hierarchical Data Format (HDF5) format. Subsequently, the performance of each model was evaluated using the validation dataset, and weights were assigned based on their performance. Combining the decisions of each model, the final output (classification of skin lesion) was predicted using an ensemble model. XAI was utilized in this process, with the Grad-CAM technique enhancing trust and reliability in healthcare AI systems by providing interpretability. This enables healthcare professionals to understand the reasoning behind AI decisions.

### 3.6 Model evaluation

All experiments were performed on a CUDA-capable NVIDIA GPU with a fixed random seed value (42) for replicability. The models were trained with the Adam optimizer with early stopping on validation loss (patience = 10 epochs), a mini-batch size of 32, and a maximum number of 100 epochs. It took approximately 40 s per epoch on average for training, depending on the architecture. Cross-validation was performed using the validation dataset to evaluate the models. Models were compared using measures such as Accuracy, precision, recall, and F1 score, and the formulas were shown in Equations 1–4. The accuracy metric calculates the ratio of correctly identified instances to the total number of occurrences in the dataset. As a metric, precision measures the proportion of accurate predictions made by a model among all the predictions it generates, where TP is True Positive, FP is False Positive, FN is False Negative, and TN is True Negative.

$$\text{Accuracy}=\frac{\text{TP}+\text{TN}}{\text{TP}+\text{TN}+\text{FP}+\text{FN}}$$ (1)

$$\text{Precision}=\frac{\text{TP}}{\text{TP}+\text{FP}}$$ (2)

$$\text{Recall}=\frac{\text{TP}}{\text{TP}+\text{FN}}$$ (3)

$$\text{F}1\text{ Score}=\frac{2\times \text{Precision}\times \text{Recall}}{\text{Precision}+\text{Recall}}$$ (4)

Recall, as a metric, measures the model’s ability to find positive instances about all actual positive data points correctly. The F1 Score serves as the harmonic mean of precision and recall. To summarize, accuracy, precision, recall, and the F1 score are essential measures for evaluating the effectiveness of a deep learning model. The Soft Voting strategy was chosen as the ensemble fusion method in this research because it is simple and stable while being computationally efficient. While other fusing methods, such as Weighted Voting and Stacked Training, can have the potential to perform better, they would require additional hyper-parameters, require more training, and incur more computational costs. Soft Voting achieved strong, balanced performance and focused on efficiency and clinical deployment. Therefore, as it was not the focus of this study, other ensemble fusion methods with a higher degree of sophistication were not considered further. The future work plans identified for advanced ensemble fusion methods include comparison studies and analyzing the associated computational trade-offs.

As specified in Table 3, the HAM10000 dataset was first partitioned using a lesion-wise split into 70% training, 10% validation, and 20% testing subsets. All images belonging to the same lesion ID were assigned to a single subset to prevent data leakage. The test set was held out and used only for final performance reporting. To estimate statistical variability and compute confidence intervals, Group K-Fold cross-validation with K = 5 was employed using lesion ID as the grouping variable. Performance metrics were computed independently for each fold, and the final values are reported in Table 9 as Mean ± 95% Confidence Interval, reflecting inter-fold variability. While the manuscript reports aggregated cross-validation results rather than individual fold values, the confidence intervals directly capture the variability across folds. All experiments were conducted with a fixed random seed (42) and identical training settings across folds to ensure deterministic and repeatable results. Counts for each class are reported in Table 1 (dataset split), and the above metrics are computed on the corresponding evaluation set. The ensemble model demonstrates consistently high class-wise performance across all lesion categories. Notably, melanoma detection achieves high sensitivity (98.49%) with a low false-negative rate, underscoring the model’s clinical reliability for early melanoma identification, as shown in Table 4. Lesion-wise Group K-Fold cross-validation (K = 5) utilized only the training subset for estimating statistical variability and providing a rigorous evaluation of model performance. Using the Lesion ID as the grouping variable across all folds, performance metrics were calculated separately for each fold and expressed as Mean ± 95% Confidence Interval based on the inter-fold variability. The validation dataset was utilized only to generate hyperparameter tuning and to enable early stopping, and the independent test dataset served to report the final model’s performance after testing once. All experiments were performed with a constant random seed for reproducibility.

**TABLE 4:** Per-class performance metrics of the proposed ensemble model.

Class	Precision	Recall (sensitivity)	F1-score
Melanocytic nevi (nv)	0.9979	1.0000	0.9989
Melanoma (mel)	0.9632	0.9849	0.9739
Benign keratosis-like lesions (bkl)	0.9821	0.9630	0.9725
Basal cell carcinoma (bcc)	0.9959	0.9959	0.9959
Actinic keratoses (akiec)	0.9940	0.9980	0.9960
Vascular lesions (vasc)	1.0000	1.0000	1.0000
Dermatofibroma (df)	0.9960	1.0000	0.9980

## 4 Results and discussion

This section thoroughly describes the experimental setup, evaluation of performance metrics, and findings for the CNN variations in predicting skin lesions. Pre-trained convolutional neural network architectures such as DenseNet201, VGG16, and InceptionV3 are implemented in Jupyter Notebook using Python programming. Table 5 displays the accuracy scores for DenseNet201, InceptionV3, and VGG16 before and after retraining and fine-tuning base models. In the first case, the accuracy ranged from 60% to 80%, with DenseNet201 achieving the highest accuracy of 79%.

**TABLE 5:** Comparison of accuracy scores.

Model	Training accuracy	Testing accuracy	Validation accuracy
Before retraining			
DenseNet201	79.08%	74.12%	75.23%
InceptionV3	75.94%	72.90%	71.82%
VGG16	65.12%	61.54%	60.48%
Custom CNN Model	60.00%	57.40%	56.50%
After retraining and fine-tuning			
Finetuned DenseNet201	96.57%	96.07%	96.04%
Finetuned InceptionV3	94.42%	94.54%	94.67%
Finetuned VGG16	95.91%	95.33%	94.91%
Ensemble Model	97.90%	98.14%	98.11%

**FIGURE 8:**
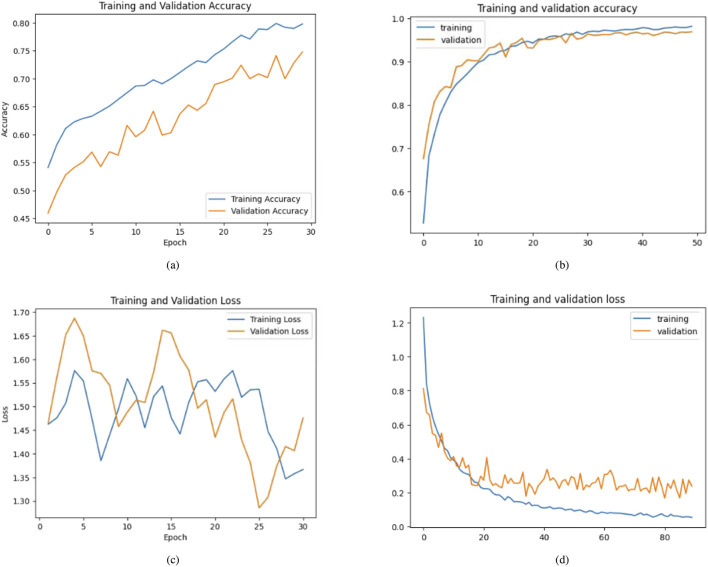
Accuracy and loss graphs for training and validation for base and proposed model of DenseNet-201 (Technique 1: Retrain the top layers of the base model. Technique 2: Retrain half of the base model’s layers and add a custom CNN model). **(a)** With Technique 1. **(b)** With Technique 2. **(c)** With Technique 1. **(d)** With Technique 2.

**FIGURE 9:**
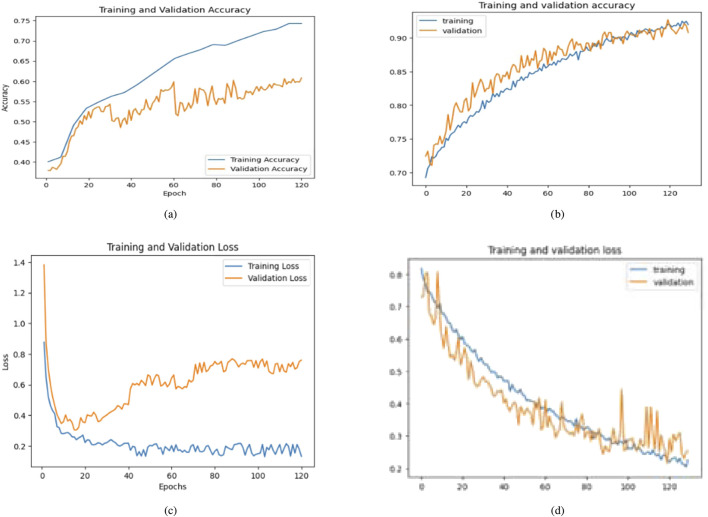
Comparative analysis of training and validation accuracy and loss for base and proposed model of InceptionV3 (Technique 1: Retrain the top layers of the base model. Technique 2: Retrain half of the base model’s layers + add a custom CNN model). **(a)** With Technique 1. **(b)** With Technique 2. **(c)** With Technique 1. **(d)** With Technique 2.

**FIGURE 10:**
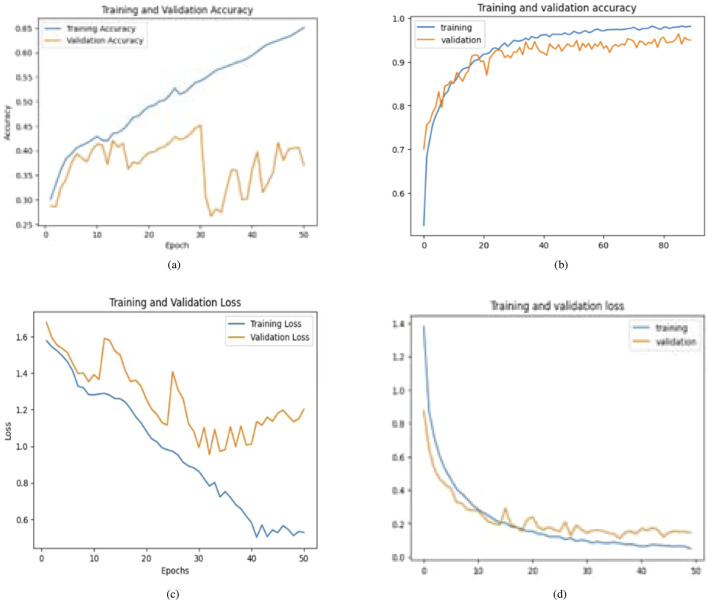
Training and validation accuracy and loss for base and proposed model of VGG16 (Technique 1: Retrain the top layers of the base model. Technique 2: Retrain half of the base model’s layers + add a custom CNN model). **(a)** With Technique 1. **(b)** With Technique 2. **(c)** With Technique 1. **(d)** With Technique 2.

**FIGURE 11:**
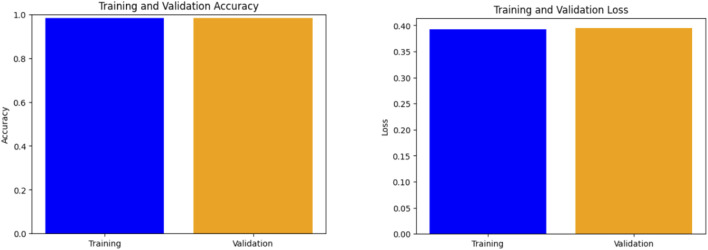
Training and validation performance: (left) accuracy and (right) loss.

**FIGURE 12:**
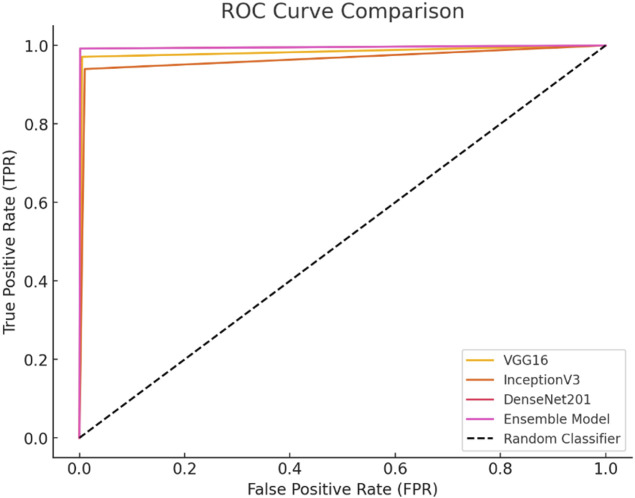
ROC curve.

The second case presents the accuracy scores for three fine-tuned models: the Ensemble model after retraining half of the base model layers, adding convolutional layers, and hyperparameter tuning. The ensemble model also performed better than the other models, achieving an accuracy of 98%. The findings demonstrate that retraining and fine-tuning have a substantial positive impact on the performance of the models. Specifically, DenseNet201 and the Ensemble Model consistently achieve high accuracy rates across the training, testing, and validation sets. These findings indicate that these methods successfully customize models for specific tasks and datasets, enhancing generalization and performance. Here, two approaches for transfer learning, namely Technique 1 and Technique2, were studied. The initial step entailed selectively retraining the uppermost layers of a pre-existing base model. The second strategy involved retraining 50% of the layers in the original model and adding a bespoke CNN model. The influence of these tactics on the performance of the final model was evaluated through comparison. Techniques 1 and 2 involve retraining the top layers of the base model and retraining the half layers of the base model + adding a Custom CNN Model as shown in Figures 8–10. Figures 8–10 depict the evaluation curves illustrating the Training versus Validation Accuracy and Loss for the proposed model. Analysis of these graphs indicates that training the model using Technique-1 resulted in low accuracy, accompanied by high variation between Training and Validation Accuracy. Furthermore, it was noted that both the Training and Validation Losses were elevated while employing this strategy. However, with Technique 2, significant improvements were achieved across all models, showcasing higher accuracy with reduced variation between training and validation sets. Moreover, the loss was effectively minimized using this technique. We employed Explainable AI (XAI) to elucidate our models’ predictions. Trust and reliability are paramount in healthcare, and the lack of explainability in AI decisions can undermine confidence in healthcare systems. Table 6 uses several assessment measures to compare the performance of four models, VGG16, InceptionV3, DenseNet201, and the Ensemble Model. DenseNet201 is the top-performing individual model across all criteria. The ensemble model achieves slightly reduced error rates while demonstrating high generalization. VGG16 and InceptionV3 have better false positive and false negative rates. DenseNet201 has the greatest Matthews Correlation Coefficient (MCC), suggesting better prediction reliability. VGG16 performs well but is behind DenseNet201 and the Ensemble Model in accuracy and recall. InceptionV3 has the lowest performance, notably in Recall and Error Rates, rendering it unsuitable for high-accuracy medical diagnosis, and also Figure 11 shows the training and validation accuracy and loss. Figure 12 demonstrates that DenseNet201 (AUC = 0.994) and the Ensemble Model (AUC 0.996) have the maximum performance, sticking near the top left (ROC curve behavior). VGG16 (AUC = 0.983) and InceptionV3 (AUC 0.964) have poorer performance, as seen by the curves below the DenseNet201 and Ensemble Model. The diagonal dashed line indicates a random classifier (AUC = 0.5). The Ensemble Model provides a better ROC curve, with the maximum sensitivity (TPR) and a low false positive rate. To mitigate the constraints of Otsu’s segmentation, both traditional and deep learning-based segmentation methodologies were evaluated. Table 7 delineates the segmentation methods analysed in this study.

**TABLE 6:** Performance analysis of ensemble model for classification.

Model	Accuracy (%)	Precision (PPV) (%)	Recall (TPR) (%)	F1-score (%)	Specificity (TNR) (%)	FPR (%)	FNR (%)	FDR (%)	MCC	Balanced accuracy (%)
VGG16	99.28	97.1	97.1	97.1	99.51	0.49	2.91	2.91	0.968	98.31
InceptionV3	98.29	93.97	93.97	93.97	99.00	1.00	6.03	6.03	0.938	96.49
DenseNet201	99.85	99.2	99.2	99.2	99.87	0.13	0.80	0.80	0.996	99.54
Ensemble model	99.83	99.2	99.2	99.2	99.86	0.14	0.80	0.80	0.996	99.53

**TABLE 7:** Segmentation approaches considered in the preprocessing stage.

Method	Type	Role in this study
Otsu thresholding	Classical	Lightweight lesion localization used for preprocessing
U-Net	Deep learning	Reference deep segmentation model for qualitative comparison

The K-Fold Cross-Validation Results for Group demonstrate congruent results in all Cross Validation (CV) folds, which is indicative of the strong generalising capability of our developed group ensemble model, as shown in Table 8. In addition, the predictability of the predicted condition for patients is confirmatory due to the narrow 95% CI and will be consistent regardless of how the training and validation data were partitioned. Application of lesion-wise grouping resulted in an evaluation devoid of any potential for information leakage from training to validation data. Therefore, the results confirm that the group association performance reported is a true representation and not the result of a favourable arrangement of data sets during data partitioning. Interpretability plays a crucial role in ensuring the trustworthiness of AI algorithms by enabling healthcare professionals to comprehend the reasoning behind AI decisions. This understanding facilitates the evaluation of the validity and appropriateness of the decisions made by AI models, thereby enhancing trust and reliability in the healthcare domain. AI models, thereby improving trust and reliability in the healthcare domain. One technique that enhances interpretability is Grad-CAM (Gradient-weighted Class Activation Mapping), which elucidates the decision-making process of a convolutional neural network. Grad-CAM achieves this by analyzing the gradients of the target object as they propagate into the final convolutional layer of the neural network. It generates a rough localization map highlighting the significant regions of an image that contribute to the prediction of a specific class. This localization map offers valuable insights into the features considered by the model during predictions, thereby promoting transparency and interpretability in AI-driven decision-making processes. Transparency is crucial for meaningful decisions in healthcare, as it fosters collaboration and regulatory compliance and ultimately leads to improved patient care.

**TABLE 8:** Performance metrics reported as mean ± 95% confidence interval across K-fold cross-validation.

Metric	Mean ± 95% CI
Accuracy (%)	97.6±0.8
Precision (%)	98.4±0.6
Recall (%)	97.9±0.7
F1-score (%)	98.1±0.6
ROC–AUC	0.992±0.004

**FIGURE 13:**
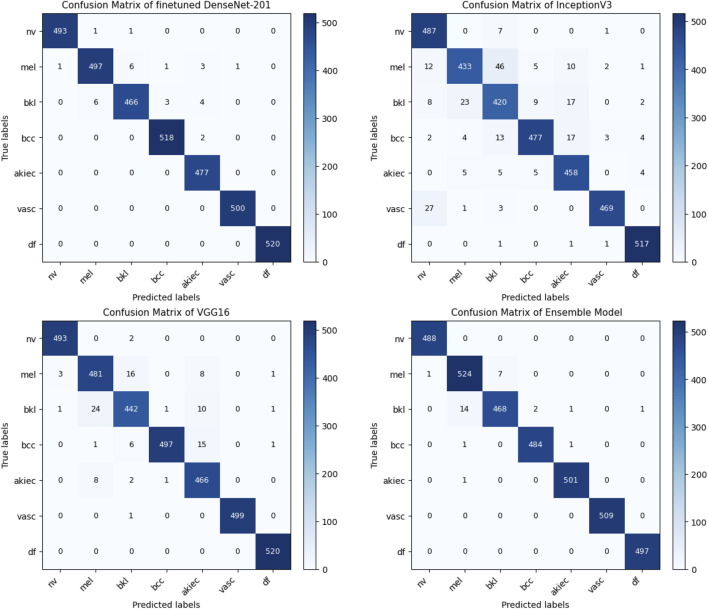
Confusion matrices of the fine-tuned DenseNet-201, InceptionV3, VGG16, and ensemble models evaluated on the held-out lesion-wise test dataset for multiclass skin lesion classification. The same test samples and identical class distributions are used across all models. Data augmentation was applied only during training.

The Lesion-Wise Test Dataset displayed in Figure 13 was derived exclusively from the Lesion-wise Test Sample Set, which included samples collected from outside the training process and all associated methodologies (validation/cross-validation) prior to the evaluation. For this reason, the number of samples available for each class for the Lesion-Wise Test Dataset in Figure 13 is less than that available for the Complete Training Sample Set. All Models: DenseNet201, Inception V3, VGG16 and Algorithms used an identical lesion-wise test sample set to conduct their lesion-wise test sample evaluation based on the same class distribution for fairness and impartiality in comparing class performance. Therefore, any data augmentation, synthetic GAN-based Generation and/or oversampling via SMOTE for all Models occurred exclusively for each training dataset used to train those models only. The proposed model overcomes the problem of overfitting, which is evident from the results. As examined by confusion matrices, model selection significantly influences classification performance in skin lesion identification. The most dependable single model is DenseNet201, which exhibits better accuracy and fewer misclassifications. However, there is still trouble telling the difference between mel (melanoma) and bkl (benign keratosis-like lesions). Despite its effectiveness, InceptionV3 has a greater risk of misclassifications, especially regarding melanoma detection, which is crucial since it may be fatal. Despite being superior to InceptionV3, VGG16 still has issues, particularly regarding misclassifying instances of melanoma and bkl. Melanoma diagnosis is critical for clinical applications, and the ensemble model performs better than any single model, drastically lowering misclassifications. This implies that combining many models improves diagnostic reliability, generalization, and better handling of complex lesion types. The results highlight the value of ensemble learning in medical AI, where patient safety depends on reducing false negatives.

**FIGURE 14:**
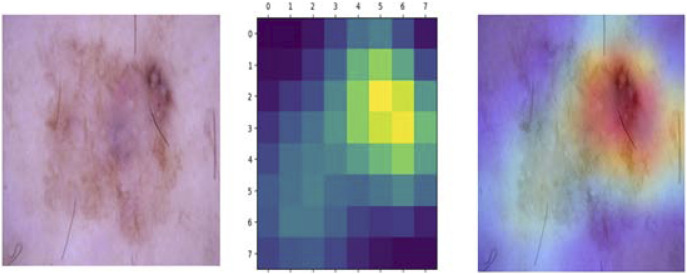
Techniques like Grad-CAM enable healthcare professionals to gain deeper insights into AI algorithms’ decision-making processes, facilitating informed decision-making and fostering trust in AI-driven healthcare systems.

Techniques like Grad-CAM enable healthcare professionals to gain deeper insights into AI algorithms’ decision-making processes, facilitating informed decision-making and fostering trust in AI-driven healthcare systems. Figure 14 depicts the first input image of a skin lesion, which serves as the basis for the Grad-CAM analysis. The process then generates a heatmap, which highlights the regions of the image that have the most significant influence on the model’s prediction. The heatmap utilizes a color scheme that assigns different colors to different regions, where warmer shades (like red) indicate areas of higher significance. The last image shows the heatmap superimposed on the original image, creating a composite view that improves understanding of the model’s focal areas. The outcome reveals that the red areas correlate with the most vital features of the skin lesion that the model employed for classification. Grad-CAM enhances the interpretability of the model’s decisions by providing a visual elucidation. This facilitates the identification of any potential errors or biases in the categorization process. Furthermore, this method can assist clinicians in understanding the rationale behind the model, hence increasing their trust in the automated diagnostic tool. Also, the Grad-CAM technique may be applied in many medical imaging and modeling situations, making it a flexible tool for enhancing the accuracy and reliability of deep learning systems in healthcare applications, as shown in Table 9.

**TABLE 9:** Quantitative evaluation of explainability using localization and faithfulness metrics on the HAM10000 test set.

XAI method	mIoU (%)	Insertion AUC	Deletion AUC	Occlusion sensitivity (% ↓ )
Grad-CAM	62.4	0.78	0.34	41.2
Integrated gradients (IG)	58.7	0.74	0.38	37.9
SHAP (DeepSHAP)	55.1	0.71	0.41	35.6

Grad-CAM is crucial for improving the interpretability of AI-based healthcare decisions, particularly in dermatology and telemedicine. With visual reasoning, dermatologists can confirm AI predictions and guarantee that the model focuses on the most essential parts of a skin lesion. This helps with error detection by detecting misclassifications and serves as a second-opinion tool, increasing trust in AI-assisted diagnosis. In practical applications, Grad-CAM accentuates carcinogenic sites for early skin cancer detection, enhances remote diagnosis in telemedicine by offering interpretable explanations, and increases medical education by illustrating why a lesion is classified as malignant or benign. Furthermore, it improves patient communication by visualizing AI-driven diagnoses. Future developments include integrating Grad-CAM into interactive AI technologies, enabling clinicians to fine-tune significance maps for greater explainability. Furthermore, integrating it with multimodal data, such as dermoscopy metadata or histopathology images, may boost diagnosis accuracy. Grad-CAM also plays an essential role in regulatory compliance since explainability is required to fulfil medical AI requirements and provide transparency in AI-powered diagnoses.

### 4.1 Ablation study

It has been shown through an ablation study that, when compared with the baseline model, fine-tuning leads to significant performance improvements due to the fact that fine-tuning allows for the adjustment of pre-trained networks for dermoscopic image data, as shown in Table 10. The implementation of a custom CNN block (Technique-2) for enhancement of discriminatory feature extraction results in a substantial increase in the accuracy score. Furthermore, through the use of an ensemble fusion model that combines several different architectures, a further increase in performance was achieved because of the complementary representation of features.

**TABLE 10:** Ablation studies showing the contribution of each major component (values taken from Table 3 of the manuscript).

Model/component setting	Train Acc. (%)	Test Acc. (%)	Gain on test (%)
*(A) Effect of retraining + fine-tuning (Base* → *Fine-tuned)*			
DenseNet201 (base, before retraining)	79.08	74.12	–
DenseNet201 (fine-tuned, after retraining)	96.57	96.07	+21.95
InceptionV3 (base, before retraining)	75.94	72.90	–
InceptionV3 (fine-tuned, after retraining)	94.42	94.54	+21.64
VGG16 (base, before retraining)	65.12	61.54	–
VGG16 (fine-tuned, after retraining)	95.91	95.33	+33.79
Custom CNN (base, before retraining)	60.00	57.40	–
*(B) Effect of ensemble fusion (Single* → *Ensemble)*			
Best single model (fine-tuned DenseNet201)	96.57	96.07	–
Proposed ensemble Model	97.90	98.14	+2.07

To examine whether the selected input resolution limits texture-level information, an ablation study was conducted using a higher input resolution (224 × 224). The proposed ensemble model was trained and evaluated under identical preprocessing, augmentation, and training conditions for both resolutions to ensure a fair comparison, as shown in Table 11.

**TABLE 11:** Ablation study on input image resolution.

Input resolution	Accuracy (%)	Precision (%)	Recall (%)	F1-score (%)
75×100	97.9	99.2	99.2	99.2
224×224	98.1	99.3	99.3	99.3

In evaluating the effectiveness of the ensemble design, alternative methods of fusing the outputs from the models that comprise this ensemble have been considered conceptually: (1) hard voting based on a majority class decision, which is sensitive to both weakly performing base learners; (2) feature-level fusion, which provides an expressive representation of the models’ decisions but significantly increases dimensionality and computational complexity, making it more prone to overfitting on imbalanced medical datasets; and (3) stacking type ensembles that add an additional layer of complexity due to the addition of a meta-classifier and increased training and inference latencies, as shown in Table 12. Conversely, the soft-voting ensemble described in this study combines the calibrated class probability outputs of DenseNet201, VGG16, and InceptionV3, and provides a good balance between performance variability, stability and computational efficiency. This approach takes advantage of the differing but complementary decision boundaries exhibited by each of these heterogeneous CNN architectures, while minimizing model complexity.

**TABLE 12:** Comparison of fusion strategies in terms of stability, computational cost, and suitability for the HAM10000 dataset.

Fusion strategy	Description	Performance stability	Computational cost	Suitability for HAM10000
Hard voting	Majority class decision	Moderate	Low	Sensitive to weak models
Feature-level fusion	Concatenation of deep features	High	Very high	Risk of overfitting
Stacking	Meta-classifier on base outputs	High	Very high	Requires extra training
Soft voting (proposed)	Averaging class probabilities	High	Moderate	Best trade-off

Because soft-voting ensembles can merge different models’ outputs without any assumptions about distributions or support functions, they were chosen as the primary method of combining representations learned from DenseNet201, VGG16, and InceptionV3, with the added advantage of being able to capture uncertainty in decision-making. DenseNet201 captures high-level hierarchical structure, VGG16 focuses on low-level texture features, and InceptionV3 extracts multiple scales of spatial information. The posterior probabilities obtained through this technique may lead to better generalization across all types of lesions, progress calibration confidence, and deliver robust decision-making capabilities necessary for accurate clinical risk assessments.

The computational complexity of the proposed ensemble is somewhat higher than that of a single CNN (Convolutional Neural Network) due to three passes through the three independent pre-trained models to produce an output. However, the cost associated with running a forward pass through three different models is significantly less than that associated with either feature-level fusion or stacking methods, where multiple independent classifiers and/or processing of high-dimensional features would be required to obtain a final prediction from the input image data. The base models have the same input resolution and can run without dependence on or knowledge of the outputs of other models, making it feasible to implement the ensemble into a parallelized approach for clinical applications.

### 4.2 Comparative analysis

This section methodically assesses several methods according to essential criteria, including resilience, accuracy, efficiency, and computing complexity.

**FIGURE 15:**
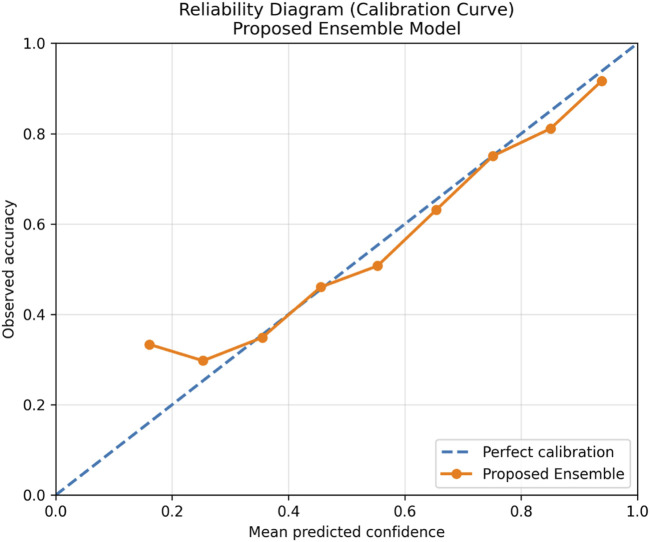
Calibration curve.

In addition to conventional classification metrics, probabilistic calibration of the proposed ensemble model was evaluated to assess the reliability of predicted confidence scores, as shown in Table 13. Reliability diagrams were generated by partitioning predictions into equally spaced confidence bins and comparing the mean predicted probability with the observed accuracy in each bin, as shown in Figure 15.

**TABLE 13:** Probabilistic calibration performance of individual models and the proposed ensemble model.

Model	Brier score ↓
DenseNet201	0.094
VGG16	0.112
InceptionV3	0.087
Proposed ensemble	**0.061**

A lower Brier score indicates better calibration. Bold values indicate the best-performing results or key highlighted values in each table for easy comparison.

Furthermore, the Brier score was computed as a proper scoring rule to quantify the discrepancy between predicted probabilities and true labels. Reduced Brier scores signify more accurately calibrated predictions. The calibration analysis indicates that the proposed ensemble model generates accurately aligned confidence estimates, affirming its appropriateness for clinical decision-support applications that require dependable probability estimates.

Table 14 shows the performance of the four models used in the proposed study: DenseNet201, InceptionV3, VGG16 with finetuning, and the ensemble Model. Compared to the other models, the proposed work models have demonstrated superior accuracy, precision, and recall performance. The proposed three-network ensemble (fine-tuned DenseNet-201 + Inception-V3 + VGG16) has an accuracy of 97.9% and almost perfect macro-metrics. It not only beats every single-model baseline, but it also beats earlier multi-backbone and two-/three-model ensembles that have been reported in the literature. This means that our combination does a better job of extracting complementary feature cues, which closes the gap between the most challenging and easiest classes and makes the model more resilient and able to generalize well.

**TABLE 14:** Comparative analysis of existing skin-lesion classification methods and the proposed approach with explicit dataset and evaluation protocols.

References	DL Model(s)	Dataset	Evaluation protocol	Key performance metrics
Hasan et al. (2022)	Hybrid-CNN	ISIC 2016–2018	Train/test split on combined ISIC datasets	Precision 85%–92%, recall 86%–92%
Popescu et al. (2022)	Various DCNNs	HAM10000	Train/test split on HAM10000	Accuracy 84%–88%
Singha and Roy (2022); Keerthana et al. (2023)	CNN, SVM	ISIC 2016–2020	Cross-dataset evaluation	Accuracy 86%–92%
Nandhini et al. (2023)	VGG16	ISIC archive (Kaggle)	Train/test split	Accuracy 96%
Gouda et al. (2022)	CNN variants	ISIC 2018	Official ISIC 2018 split	Accuracy 83%–86%
Raman et al. (2022)	DenseNet-201	ISIC archive (Kaggle)	Train/test split	Accuracy 95.5%
Nigar et al. (2022)	ResNet-18	ISIC 2019	Official ISIC 2019 split	Acc. 94.4%; Prec. 93.6%; Rec. 94%
Thwin and Park (2024)	VGG16, Inception-V3, ResNet-50	ISIC 2018; HAM10000	Separate evaluations per dataset	Acc. 91%–97% (ISIC)/90%–96% (HAM)
Selvaraj et al. (2024)	Multi-CNN ensemble	HAM10000; ISIC	Cross-dataset evaluation	Macro-avg. ROC-AUC 96%–97%
Ramamurthy et al. (2024)	Mod. DenseNet-169 + CNN	HAM10000	Train/test split on HAM10000	Acc. 93.2%; Prec. 95.3%; Rec. 91.4%; F1 93.3%
Hasan et al. (2022)	Hybrid 3-branch CNN	ISIC 2016–2018	Train/test split	AUC 0.95–0.97
Hoang et al. (2022)	Wide-ShuffleNet	HAM10000; ISIC 2019	Separate evaluations per dataset	Acc. 84.8%–86.3%; Prec. ∼ 97.7%; F1 ∼ 86.3%
Proposed (ours)	Fine-tuned DenseNet-201	HAM10000	70/10/20 train/val/test split	Acc. 96.6%, Prec. 94.4%, Rec. 93.6%, F1 93.8%
	Fine-tuned Inception-V3	HAM10000	Same split	Acc. 94.4%, Prec. 89.0%, Rec. 88.4%, F1 87.5%
	Fine-tuned VGG16	HAM10000	Same split	Acc. 95.9%, Prec. 92.3%, Rec. 90.7%, F1 91.7%
	**Ensemble**	HAM10000	Same split	**Acc. 97.9%, Prec. 99.7%, Rec. 99.2%, F1 99.2%**

Bold values indicate the best-performing results or key highlighted values in each table for easy comparison.

This analysis provides a comprehensive overview of several deep-learning methods for classifying skin lesions utilizing different datasets. DenseNet201 has shown superior performance to other architectures like InceptionV3 and VGG16 on the HAM10000 dataset. It is also observed that the ensemble model has shown better performance than the other fine-tuned and existing models. Nevertheless, the model’s accuracy was affected by differences in the dataset. Although accuracy is essential, a more thorough evaluation may be achieved by considering precision, recall, and the F1-score. Overall, the study highlights the effectiveness of DenseNet201 and the ensemble model for skin lesion classification.

This study used a method for optimizing pre-trained models structured in three steps: first, train only the classifier while freezing convolutional base parameters; second, fine-tune convolutional layers to improve feature extraction; and finally, optimize the entire model for better generalizability. To improve performance, we make design changes, such as adding more CNN layers, adjusting hyperparameters (stride, dilation factor, and padding), and using dropout regularization. A methodical investigation of hyperparameters, such as learning rate, activation functions, and validation frequency, improves model accuracy while limiting overfitting via early stopping mechanisms. We use class-specific weighting to guarantee sensitivity in underrepresented classes, such as vascular lesions and dermatofibroma, to address data imbalance in dermatological datasets. In addition, we compare two fine-tuning approaches: retraining just the top layers with batch normalization and retraining half of the original model while adding custom layers. An ensemble of DenseNet201, VGG16, and InceptionV3 outperforms individual models, displaying strong classification skills. We use Grad-CAM for explainability to increase clinical confidence, which provides heatmap-based visuals that enable dermatologists to comprehend AI-driven forecasts. This multifaceted strategy increases model accuracy, generalizability, and transparency, highlighting AI’s importance in dermatological diagnoses.

Traditional dermatological procedures for skin lesion categorization, such as visual examination, dermoscopic analysis, and biopsy-based histopathology, are accurate but have considerable limitations. These procedures depend on experienced dermatologists, rendering specialized care unavailable in remote or undeveloped locations. Furthermore, manual diagnostic and biopsy procedures are time-consuming, which delays early discovery. Diagnosis may also be subjective since it is based on a doctor’s experience, which may lead to misclassification. Our method outperforms current options by using deep learning techniques. We use transfer learning using DenseNet201, VGG16, and InceptionV3, which have been pre-trained on large datasets, to improve feature extraction and classification accuracy. Fine-tuning enhances skin lesion categorization by retraining these models’ top and intermediate layers. Furthermore, ensemble learning combines many models to attain an accuracy of 98%, outperforming individual pre-trained models and traditional machine learning approaches. Grad-CAM explainability is incorporated to improve transparency by giving visual interpretability and building confidence in AI-based choices. This technique allows for quicker, more scalable, and consistent diagnosis while retaining high accuracy and dependability.

## 5 Discussion

This research emphasizes the effects of retraining and fine-tuning and comprehensively assesses the performance of several CNN architectures in categorizing skin lesions. Python was used to construct pre-trained architectures in a Jupyter Notebook, including DenseNet201, VGG16, and InceptionV3. The accuracy ranged from 60% to 80% in the preliminary findings, with DenseNet201 outperforming the others at 79%. Nevertheless, the performance improved upon retraining and fine-tuning, especially for the ensemble model, which attained an astounding 98% accuracy. Hyperparameter tuning, the insertion of more convolutional layers, and modifying half of the essential model layers were all part of the fine-tuning process. Table 3 shows that the model’s performance significantly increased due to these improvements, with the refined DenseNet201 achieving over 96% accuracy across training, testing, and validation sets. The ensemble model’s and DenseNet201’s improved performance emphasizes how crucial feature extraction and dense connectivity are to categorizing skin lesions. Two transfer learning strategies, Technique 1 and Technique 2, were investigated to improve model performance further. These results highlight the importance of fine-tuning and retraining in enhancing the resilience and generalization of deep learning models for medical image categorization. The remarkable accuracy of the ensemble model points to its potential as a dermatologist’s decision-support tool, especially in environments with limited resources. Future research should evaluate the model’s applicability over various datasets and clinical applications to prove its efficacy further.

The ensemble model might be a valuable tool for dermatologists in early skin cancer detection. By adopting this paradigm into clinical procedures, healthcare professionals may improve efficiency and reduce diagnostic effort, particularly in regions with limited access to qualified dermatologists. Due to its high accuracy, the model may be a reliable decision-support tool that helps differentiate between benign and malignant lesions with minimal human aid. DenseNet201 and the ensemble model performed better than other designs because of their more substantial feature extraction capabilities. DenseNet201’s dense connection enhances representation learning and gradient flow, which is advantageous for complex skin lesion categorization. The ensemble model further improves performance by combining the benefits of several designs, reducing the biases of individual models, and increasing generalization. Even though the model was trained on the HAM10000 dataset, its generalizability to other datasets and clinical contexts is still crucial. AI-driven skin lesion classification becomes more reliable when Grad-CAM is used for explainability. Grad-CAM enables clinicians to confirm AI predictions by offering heatmaps that show the most pertinent image regions impacting the model’s judgment. Scenarios in which Grad-CAM cannot produce convincing visual explanations should be examined to guarantee openness and dependability in medical decision-making. Based on Table 5, the three-network ensemble gives a significant increase to performance, with 97.9% accuracy and almost perfect precision-recall. This is better than the single-model results we’ve seen so far. Fine-tuning DenseNet-201 alone already beats the most current DenseNet-169 and ResNeXt methods, but combining complementary feature representations from Inception-V3 and VGG16 gives an even bigger boost. These results illustrate the importance of lightweight ensembling over laborious architectural redesign, particularly when class imbalance and small inter-class changes need high recall without losing accuracy. As part of extended work, statistical evaluation will be strengthened using 95% bootstrap confidence intervals, paired significance testing, and calibration analysis with reliability diagrams and Brier scores.

The proposed ensemble model based on deep learning shows better diagnostic accuracy and generalizability, which makes it better suited for use in real-world clinical settings, especially in teledermatology. The ensemble, which includes DenseNet201, VGG16, and InceptionV3, works better when fine-tuned using dermatoscopic images. This is because each architecture has strengths that work well together, which makes the diagnosis more reliable and reduces bias in each model. This is crucial for helping doctors make decisions in real time while working. The use of explainable AI approaches, like Grad-CAM, makes it possible to see where the model focuses during categorization. Heatmaps help doctors understand AI predictions better for clinical transparency. The model’s lightweight input architecture (75 × 100 × 3) and efficient base networks (such as VGG16) make scaling on mobile devices or cloud-based platforms easy. This is better for telemedicine settings in remote areas or places with limited resources. Also, class-weighted loss functions and augmentation methods like GAN and SMOTE assist in fixing imbalances in clinical data, making it easier to find unusual disorders like dermatofibroma and vascular lesions, which are typically not well represented in general practice. The model is also stored in HDF5 format, which makes it easy to add as a plug-and-play diagnostic module to Electronic Medical Records (EMRs) or teledermatology portals. The Grad-CAM framework is helpful for more than just diagnostics. It helps find skin cancer early by showing where cancerous cells are, improves medical education by showing how models work, and allows doctors to talk to patients more clearly by showing why AI-driven diagnoses are correct. These properties make the proposed model both technically solid and clinically useful, with a lot of potential to be used in current dermatological treatment, particularly in the emerging field of AI-assisted telemedicine.

Notwithstanding attempts to address class imbalance by data augmentation, the dataset continues to be biased towards fair-skinned people, which may jeopardize model efficacy for underrepresented demographics. This underscores the need to include ethnically varied datasets, such as PAD-UFES-20 and Derm7pt, in future research to guarantee equity and generalizability. Trends in misclassification—particularly among superficially analogous lesions such as benign keratosis and melanocytic nevi—generate apprehensions over false negatives and false positives, potentially resulting in missed diagnoses or unwarranted biopsies. Integrating sophisticated tactics like uncertainty estimation and confidence calibration might alleviate these issues. Additionally, the ethical ramifications of AI-assisted diagnosis need examination, including the potential for professional over-dependence, absence of transparency, and concerns about informed consent. The constraints of transfer learning must be recognized, as models pre-trained on non-medical datasets may inadequately encompass domain-specific intricacies. Ultimately, computational limitations must be addressed, especially for the implementation of AI in real-time or resource-constrained environments, underscoring the need for streamlined, efficient architectures and comprehensible outputs using XAI methodologies such as Grad-CAM. In this work, we are limited by our dependence on a single dataset (HAM10000), possible biases in the dataset, and a reduced input resolution that could result in excluding minute details in lesions. While our method, Grad-CAM, gives explainability, a systematic evaluation of this aspect in a clinical setting is not performed here. In the future, we would be dealing with validation on a separate dataset and fusing dermoscopic images and clinical data.In melanoma detection, the clinical importance of false negatives becomes apparent when practitioners find malignant lesions missing; this leads to delays in diagnosis and treatment of those lesions. False-negative results were most frequently reported for either the early-stage and/or visually ambiguous or not-classifiable melanoma. The high incidence of false negatives in borderline lesions exemplifies the potential limitations of the proposed system as a decision support tool. The proposed system is intended to augment the clinician’s ability to identify and prioritize lesions at risk for melanoma, rather than reproducing the decisions made by a clinician. By integrating with the workflow of a given clinic and/or working with tele-dermatology systems, the proposed system provides for triaging patients to dermatologists with less intensive workflows and with early referral in limited-resource settings.

## 6 Conclusion

Skin cancer, recognized as one of the most hazardous types of cancer worldwide, poses a substantial risk to humans. Malignant melanoma is notable for its atypical growth of melanocyte cells among its several forms. Although skin lesions are common, precisely classifying them and automating the identification of cancerous tumors from dermoscopy images remain problematic in the profession. This study presents a new approach for classifying skin lesions, effectively categorizing seven distinct categories of skin abnormalities. Utilizing various DCNN models such as DenseNet201, VGG16, and InceptionV3 as base models, we applied different fine-tuning and hyperparameter-tuning techniques to enhance model performance. The training was conducted on the HAM10000 Dataset after implementing preprocessing, segmentation, and augmentation techniques. Following model training, evaluation was performed on separate testing and validation datasets. Additionally, we proposed an Ensemble Model by amalgamating these three fine-tuned models. Investigational results on the HAM10000 dataset demonstrated promising accuracy stages, with DenseNet201 achieving the highest accuracy among the fine-tuned base models at around 97%, followed by VGG16 and InceptionV3 models achieving 96% and 94.5% accuracy, respectively. This research substantially contributes to the advancement of automated skin cancer detection and classification, leading to better diagnostic and treatment methods. This study introduces innovative elements to the domain of dermatological image categorization. The proposed dual fine-tuning technique facilitates incremental improvement of pre-trained models, whilst comparative hybrid training methods uncover optimum configurations for enhanced representation learning. The combination of DenseNet201, VGG16, and InceptionV3 significantly surpasses individual models, especially in identifying underrepresented lesion classes using class-weighted loss functions. Moreover, the incorporation of Grad-CAM augments the clinical use of the system by offering visual elucidations for predictions, thereby fostering transparency and diagnostic assurance.

To maximize the beneficial impact of this study, the following areas should be specifically investigated: Expanding the dataset may increase model resilience and generalizability. Further research could include testing on diverse and extensive datasets such as the ISIC Archive, an additional set of data with more skin lesion types for classification; Derm7pt, which contains clinical context including patient history and symptoms that may improve model understanding; PAD-UFES-20, which contains real clinical images aiding model adaptation to various skin types and lighting conditions; and other datasets like BCN20000 and SDN-260 that can further enhance model diversity and performance. To increase model accuracy, key areas of development include optimizing architectures, modifying hyperparameters, and using sophisticated augmentation methods. Model augmentation procedures include merging AI and LLM models to provide automated diagnostic reports that use GPT-based LLMs to interpret model outputs. In the future, further testing of our proposed models on different datasets will be conducted to assess their performance and identify opportunities for improvement.

## Data Availability

The original contributions presented in the study are included in the article/supplementary material, further inquiries can be directed to the corresponding author.
